# A New Option for Pain Prevention Using a Therapeutic Virtual Reality Solution for Bone Marrow Biopsy (REVEH Trial): Open-Label, Randomized, Multicenter, Phase 3 Study

**DOI:** 10.2196/38619

**Published:** 2023-02-15

**Authors:** Katell Le Du, Anne-Lise Septans, Frédéric Maloisel, Hélène Vanquaethem, Anna Schmitt, Marielle Le Goff, Aline Clavert, Marie Zinger, Hugues Bourgeois, Olivier Dupuis, Fabrice Denis, Stéphane Bouchard

**Affiliations:** 1 Department of Hematology Confluent Private Hospital Nantes France; 2 Department of Onco-Hematology Institut inter-régionaL de Cancérologie Centre Jean Bernard Le Mans France; 3 Department of Onco-Hematology Clinique Saint-Anne Strasbourg France; 4 Department of Internal Medicine Hôpital d’Instruction des Armées de Bégin Saint-Mandé France; 5 Department of Hematology Institut Bergonié Comprehensive Cancer Center Bordeaux France; 6 Department of Hematology Centre Hospitalo-Universitaire Angers France; 7 Department of Onco-Hematology Clinique Victor Hugo Le Mans France; 8 Department of Psychoeducation and Psychology Université du Québec en Outaouais Gatineau, QC Canada

**Keywords:** virtual reality, VR, bone marrow, biopsy, pain, digital therapeutics, digital health, eHealth, RCT, randomized controlled trial, clinical trial, distraction, imagery, imagination, imaginary, immersive environment, interactive environment, head-mounted display, medical procedure, satisfaction, safety, efficacy, effectiveness

## Abstract

**Background:**

Evidence regarding the analgesic effect of distraction through immersion in virtual reality (VR) for care-induced pain has been documented in several phase 2 trials, but comparison with standard treatments in large, randomized studies is needed.

**Objective:**

In this open-label, multicenter, randomized, phase 3 trial, we evaluated the safety and efficacy of a novel VR therapy solution for distraction in the context of bone marrow biopsy.

**Methods:**

Bliss is a VR software with 4 imaginary interactive environments in 3 dimensions with binaural sound (head-mounted display). Efficacy regarding pain intensity was evaluated using a visual analog scale (VAS; score from 0 to 10) immediately after the biopsy. Secondary end points were anxiety and tolerance. Modified intention-to-treat analysis was performed.

**Results:**

Overall, 126 patients with previously documented untreated or suspected malignant hemopathy between September 6, 2018, and May 18, 2020, were randomly assigned in a 1:1 ratio to receive pain prevention with a mixture of nitrous oxide/oxygen (MEOPA; n=63) or VR (n=63) before and during the bone marrow biopsy. We excluded 8 patients from the final analysis (3 in the MEOPA group and 5 in the VR group). All patients received local anesthesia (lidocaine) before biopsy. Follow-up was limited to 1 month after the biopsy. Participants’ median age was 65.5 (range 18-87) years, and 54.2% (64/118) of patients were male. The average pain intensity was 3.5 (SD 2.6, 95% CI –1.6 to 8.6) for the MEOPA group and 3.0 (SD 2.4, 95% CI –1.7 to 7.7) for the VR group, without any significant differences in age, sex, center, and hemopathy (*P*=.26). Concerning anxiety, 67.5% (79/117; fear of pain questionnaire) of the patients were afraid before the biopsy, and anxiety scores were moderate to very high in 26.3% (30/114; revised Spielberger State-Trait Anxiety Inventory questionnaire) of the patients before the biopsy and 9.0% (10/114) after the biopsy for all patients, without a significant difference between the 2 groups (*P*=.83). Immersion in VR was well tolerated by the majority (54/57, 95%) of patients in the VR group.

**Conclusions:**

The intensity of pain did not significantly differ between both arms. VR was well tolerated, and the satisfaction of patients, nurses, and physicians was very high. VR could be an alternative treatment in case of contraindication or intolerance to MEOPA.

**Trial Registration:**

ClinicalTrials.gov NCT03483194; https://clinicaltrials.gov/ct2/show/NCT03483194

## Introduction

Bone marrow biopsy is a routine procedure for the exploration of hematologic disorders and malignant hemopathies. Biopsy is generally performed on the anterior or posterior iliac crest, and despite local analgesia with lidocaine, the pain level still remains high [1]. A long procedure duration (>30 minutes), limited operator experience, high BMI, and advanced age have been reported to increase levels of pain [1-3]. Pain is also influenced by the emotional status of the patient before the biopsy, and a high level of anxiety can increase visual analog scale (VAS) scores [4]. Median pain scores have been published previously under standard conditions (ie, with local anesthesia only) and ranged from 1.9 with VAS to 3.0 with a numeral rating scale [5,6]. Several oral or intravenous drugs (oxycodone, tramadol, diazepam, lorazepam, and midazolam) that have already been tested to reduce pain and anxiety [7-10] are not easy to use with outpatients, and secondary effects must be monitored (amnesia, nausea, dizziness, and loss of vigilance) [11,12]. An inhaled mixture of nitrous oxide/oxygen (MEOPA) is easier to use for patients, nurses, and physicians and has become standard to prevent pain despite a lower efficacy [13,14] and some side effects (hypoxia, nausea, vomiting) [15,16]. However, MEOPA has been requested by patients for further biopsies, especially for children [7,17]. Other nonpharmacologic treatments (music therapy, hypnosis, and behavioral therapy) have been assessed through clinical trials; anxiety levels, but not pain intensity, were significantly reduced [18-20]. Digital therapeutics have recently emerged, and immersion in virtual reality (VR) environments has demonstrated efficacy in preventing pain and anxiety in phase 2 trials [21-27]. A more recent study was conducted with patients undergoing sternal bone marrow aspiration with subcutaneous lidocaine, with or without immersion in a VR program: no difference was observed in pain and anxiety scores [25]. None of these studies compared MEOPA or other preventive treatments with a VR program. The REVEH trial assessed the efficacy and safety of a new immersive VR method for pain prevention, in comparison with MEOPA.

## Methods

### Study Design

We conducted an open-label, prospective study between September 6, 2018, and May 18, 2020, in 5 centers in France.

### Ethical Considerations

The trial was conducted in accordance with the Declaration of Helsinki of 1975, as revised in 2008, and the International Conference on Harmonization Good Clinical Practice guidelines for biomedical research. The “Sud-Méditerranéen I” Regional Ethics Committee approved the study on February 14, 2018 (2017-A02701-52), and the Agence Nationale de Sécurité du Médicament approved it on March 9, 2018. All patients provided written informed consent.

### Study Population and Inclusion and Exclusion Criteria

Eligible patients were aged 18 years and older with previously documented or suspected untreated malignant hemopathy with an indication for a bone marrow biopsy. An Eastern Cooperative Oncology Group (ECOG) performance status between 0 and 2 and normal biological coagulation parameters were required. Clinical examination and bone pelvic imaging were performed before inclusion to exclude iliac lymphoma localization. The exclusion criteria were pregnancy, congenital or acquired coagulation deficit, thrombocytopenia less than 50,000/mm^3^, and use of certain drugs (fluindione, acenocoumarol, warfarin, dabigatran, apixaban, rivaroxaban, and analgesics for chronic pain). We also excluded patients for whom MEOPA was not recommended (intracranial hypertension, allergy, severe lung failure with oxygen therapy, emphysema, pneumothorax, recent history of air embolism, epilepsy, and vitamin B12 and B9 deficiencies) and those in whom 3-dimensional movies (pacemaker or defibrillator) were contraindicated.

### Randomization

Randomization was planned by minimization upon inclusion of patients in the study and programmed using ENNOV clinical data management software (ENNOV; Paris, France). Patients were randomly assigned 1:1 to a pain prevention program using MEOPA or VR. Treatment allocation was stratified by center, age, sex, and hemopathy. In case of intolerance in the VR arm, a change to MEOPA was permitted.

### Primary and Secondary End Points

The primary endpoint was a pain reduction of 1.5 points on the VAS for patients included in the VR group in comparison with the MEOPA group. The secondary objectives were the number of lidocaine vials used; duration of exposure to MEOPA (not evaluated in the VR arm); tolerance of the VR session; anxiety level before and after the biopsy; sense of presence for the VR arm; level of residual pain and memory of pain after 1 month of follow-up; and assessment of patient, nurse, and physician satisfaction.

### Bone Marrow Procedure

All patients were lying face down with their hands positioned under the head (based on the French Society of Hematology guidelines) [28]. Local anesthesia was performed with a lidocaine injection (1 vial containing 20 mL had a concentration of 10 mg/mL). Biopsy was then performed on the posterior iliac crest with a classic trocard (Jamshidi or Monoject bone marrow biopsy needle).

In the MEOPA group, administration was started at the same time as the local anesthesia. In the VR group, a 5-minute demonstration session was proposed on the day of randomization to assess tolerance before the biopsy, and the program was started 5 minutes before anesthesia with a maximum duration of 40 minutes. In cases of intolerable pain during the procedure, salvage treatment with a second local injection of lidocaine and/or paracetamol (1 g) or alprazolam (0.25 mg) was proposed.

### Description of VR Programs

**Figure 1:**
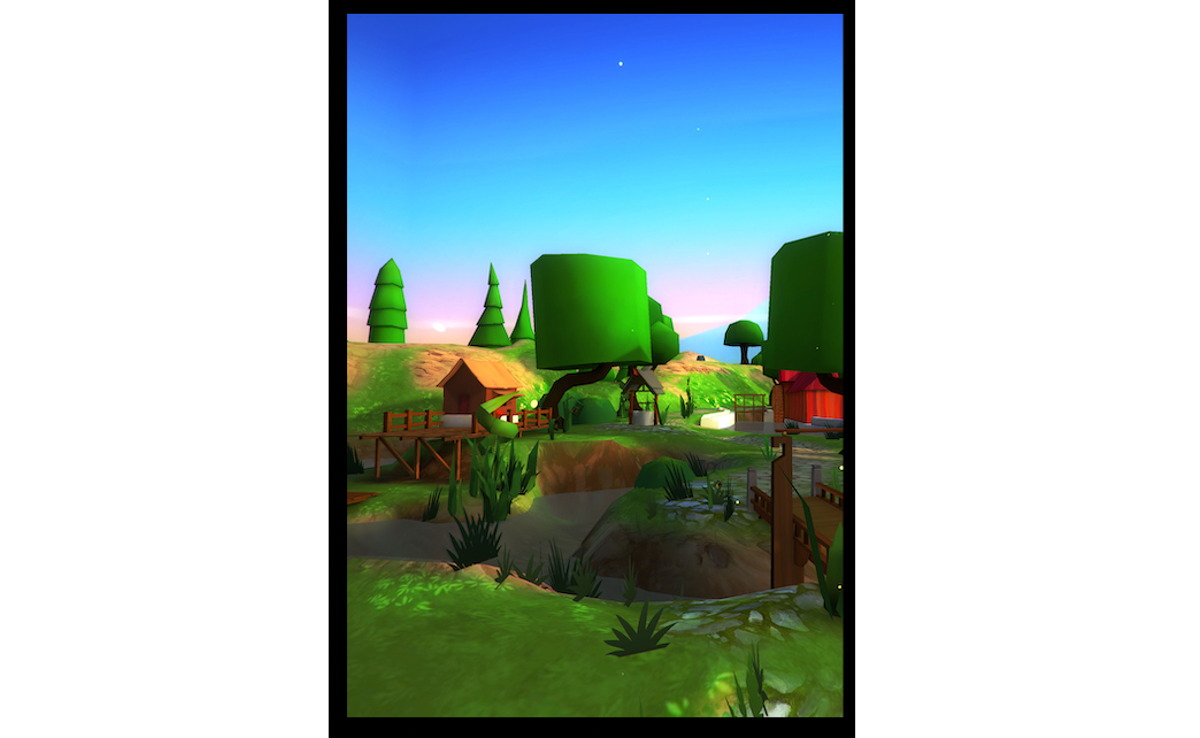
Nohara environment.

**Figure 2:**
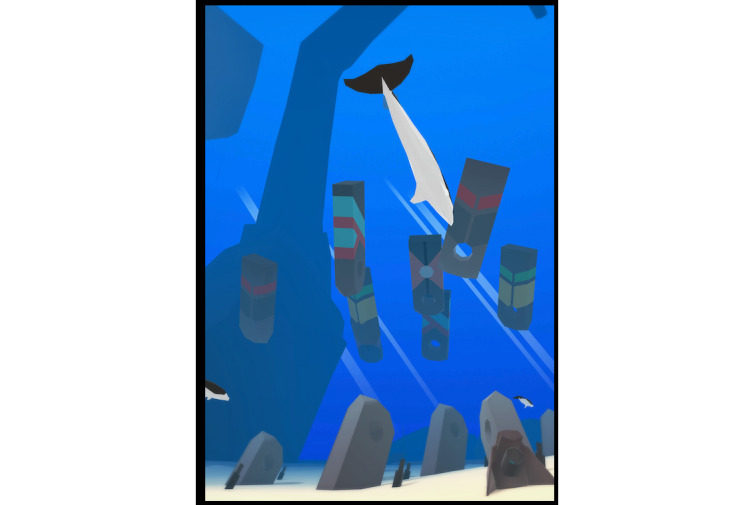
Kaetei environment.

**Figure 3:**
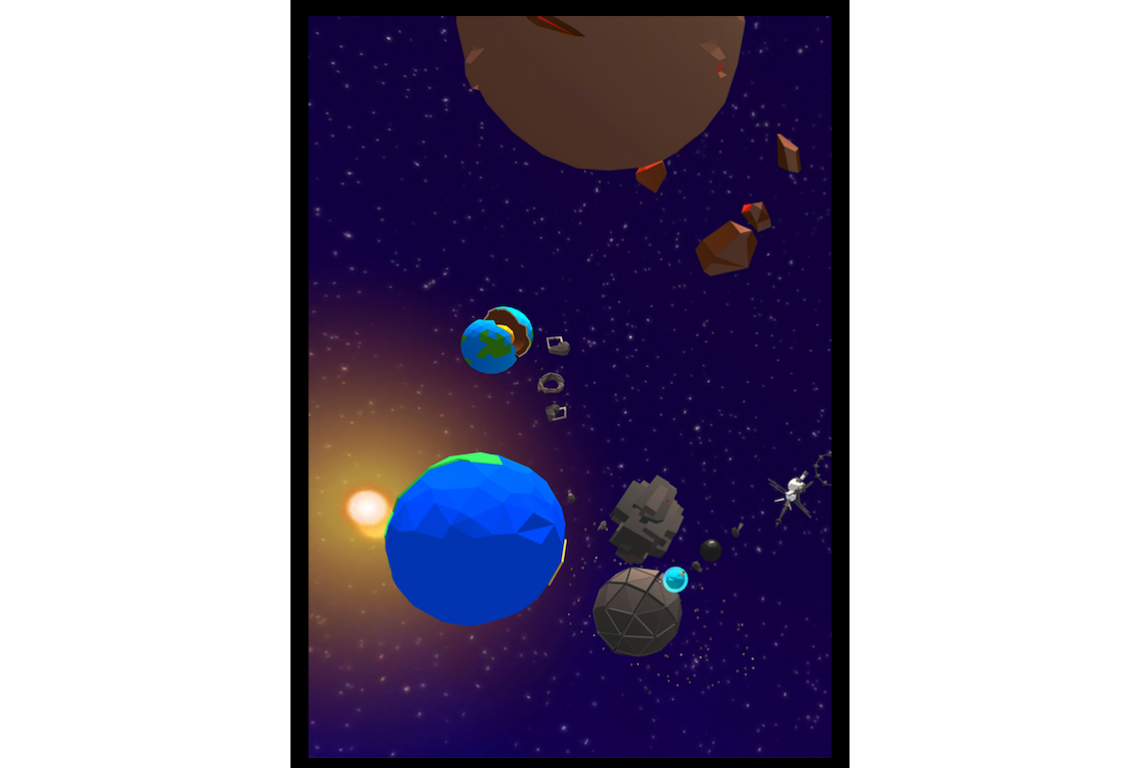
Uchuu environment.

**Figure 4:**
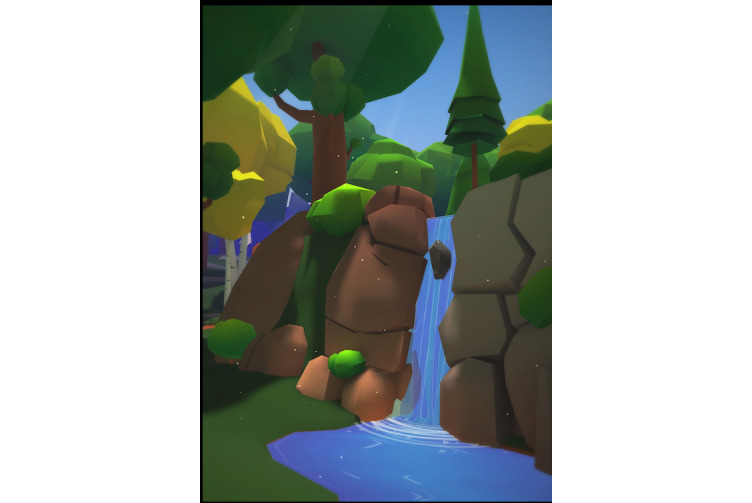
Mori environment.

Bliss is a type 1 medical device with European accreditation (EN 50581: 2012; manufacturer: Effet Papillon Company, Laval, France). It is a 3-dimensional interactive VR application. The program runs on a smartphone and a GearVR head-mounted display. We proposed 4 imaginary VR environments with a median duration of 15 minutes to 40 minutes: Nohara (dream-like walk on the country side), Kaitei (seabed exploration), Uchuu (space walk), and Mori (dream-like walk in the forest; Figures 1-4). All these environments were designed to induce a state of relaxation and light sedation through slow passive contemplative exploration without inducing a hypnotic state. For total immersion, the patients wore headphones with binaural sound. Synchronous sounds in the virtual environment contributed to stereophony and immersion in a 3-dimensional environment by increasing the concentration and eliminating other sources of noise. The soundtrack was specific to each program and recorded by musicians and sound engineers for the trial. A complete kit was made available at each study center. All parts of the kit were reusable and cleaned between patients.

### Data Recorded the Day of the Biopsy

Blood pressure was measured before the biopsy and 15 minutes after the biopsy. Anxiety was assessed using 2 questionnaires: a local fear of pain questionnaire before the biopsy (presence of fear, intensity of fear by VAS, and causes of fear) and the adapted Spielberger State-Trait Anxiety Inventory (STAI) form Y before and 15 minutes after the biopsy [29]. The STAI score ranges between 20 and 80 points: the higher the score, the more anxious the patient. We defined 5 categories (very low: <36; low: 36-46; average: 46-56; high: 56-65; very high: >65). Nausea, headache, and dizziness were recorded to assess tolerance in each arm. The sense of presence (feeling of being immersed) was assessed for the VR group using 3 questions (feeling of being immersed, dreaming, and escaping). Nurses and the investigators registered pain intensity 15 minutes after the procedure using the VAS. Satisfaction of the patients, nurses, and doctors was assessed at the end of the biopsy.

### Data Recorded 1 Month After the Biopsy

The level of residual pain and the memory of pain were assessed by the investigators 1 month after the biopsy (responses: yes or no; if yes, the intensity level was assessed using a VAS).

### Statistical Analysis

The primary end point was to measure the intensity of pain in each group using the VAS and to demonstrate a 1.5-point reduction in pain in the VR group. We enrolled 120 patients to validate this hypothesis with an SD of 2.5, power of 90%, and type I error rate of 5%. We included 6 more patients, considering that 5% could not be evaluated using 2-tailed tests. Intention-to-treat analysis included patients for whom pain assessment was available.

Variables are presented in tables; the Student *t* test was used to compare quantitative data, and the chi-square test or Fisher exact test was used to assess qualitative data. Correlation analysis was performed using the Spearman test.

All statistical analyses were conducted using SAS 9.3 software (SAS Institute Inc) with a significance of 5%.

## Results

### Sample

**Figure 5:**
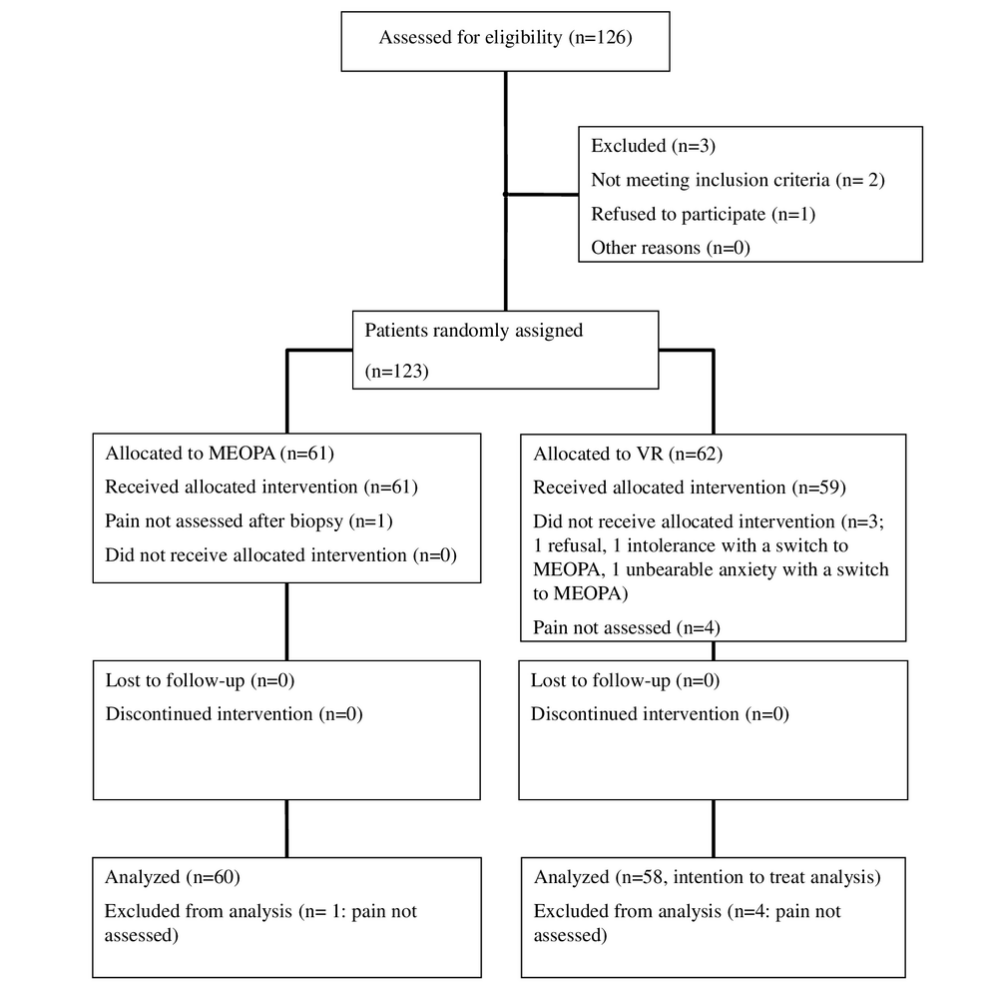
CONSORT diagram. MEOPA: mixture of nitrous oxide/oxygen; VR: virtual reality.

A total of 126 patients were enrolled between September 6, 2018, and May 18, 2020, in 5 centers in France: Le Mans (n=80), Strasbourg (n=32), Bordeaux (n=8), Saint-Mandé (n=5), and Angers (n=1). Trial recruitment stopped after inclusion of the target population. We excluded 3 patients before randomization (2 patients did not meet the inclusion criteria, and 1 patient withdrew consent), and 5 patients were excluded after randomization from the final analysis (intensity of pain not documented: 4 patients in the VR group and 1 patient in the MEOPA group). An additional 3 patients did not complete the VR session in the VR group: 1 patient refused VR after the short demonstration and then also refused MEOPA, 1 patient was intolerant, and 1 patient experienced unbearable anxiety. The 2 latter patients both received MEOPA during the biopsy. They were excluded from the per-protocol analysis but included in the intention-to-treat analysis (Figure 5).

### Patient Characteristics

The median age of the study population was 65.5 (range 18-87) years, and 54.2% (64/118) were male. Most (105/118, 89.0%) of the patients were ECOG 0-1. The 2 groups were equally balanced in terms of age (*P*=.68) and sex (*P*=.84). Lymphoma and myeloproliferative disorders were the most common diagnoses (63/118, 53.4%, and 26/118, 22.0%, respectively). Blood pressure was recorded for 116 patients before the biopsy (54 in the MEOPA group and 52 in the VR group; *P*=.63) and 92 patients after the biopsy (46 in the MEOPA group and 46 in the VR group; *P*>.99). The demographics and patient characteristics are shown in Table 1.

**Table 1:** Patient characteristics.

Characteristics			Total (n=118)		MEOPA^a^ (n=60)		VR^b^ (n=58)		*P* value^c^	
Age (years), median (range)			65.5 (18-87)		66 (18-87)		66 (38-87)		.68	
**Sex, n (%)**										.84
	Male	64 (54.2)		32 (53.3)		32 (55.2)				
	Female	54 (45.8)		28 (46.7)		26 (44.8)				
**ECOG^d^, n (%)**										.80
	0	53 (44.9)		29 (48.3)		24 (41.4)				
	1	52 (44.1)		24 (40.0)		28 (48.3)				
	2	5 (4.2)		3 (5.0)		2 (3.4)				
	Unknown	8 (6.8)		4 (6.7)		4 (6.9)				
**Diagnosis, n (%)**										N/A^e^
	Lymphoma	63 (53.4)		29 (48.3)		34 (58.6)				
	Myeloproliferative neoplasms	26 (22.0)		14 (23.3)		12 (20.7)				
	Myelodysplastic syndrome	4 (3.4)		3 (5.0)		1 (1.7)				
	Leukemia	6 (5.1)		3 (5.0)		3 (5.2)				
	Myeloma	5 (4.2)		3 (5.0)		2 (3.5)				
	Monoclonal gammopathy	4 (3.4)		2 (3.3)		2 (3.5)				
	Solid tumor	1 (0.9)		1 (1.7)		0 (0)				
	Other	9 (7.6)		5 (8.3)		4 (6.9)				
**Initial clinical exam, n (%)**										>.99
	Not done	6 (5.1)		3 (5.0)		3 (5.2)				
	Done	112 (94.9)		57 (95.0)		55 (94.8)				
**Initial iconography, n (%)**										.55
	Not done	83 (70.3)		41 (68.3)		42 (72.4)				
	Done	34 (28.8)		19 (31.7)		15 (25.9)				
	Unknown	1 (0.9)		0 (0)		1 (1.7)				
**First blood pressure measurement (15 minutes before the biopsy), n (%)**										.64
	Not done	10 (8.6)		4 (6.8)		6 (10.3)				
	Done	106 (89.8)		54 (90.0)		52 (89.7)				
	Unknown	2 (1.7)		2 (3.3)		0 (0)				
**Second blood pressure measurement (15 minutes after the biopsy), n (%)**										>.99
	Not done	24 (20.3)		12 (20.0)		12 (20.7)				
	Done	92 (77.9)		46 (76.7)		46 (79.3)				
	Unknown	2 (1.7)		2 (3.3)		0 (0)				

^a^MEOPA: mixture of nitrous oxide/oxygen.

^b^VR: virtual reality.

^c^Chi-square test, except for age, which was analyzed using a Wilcoxon test.

^d^ECOG: Eastern Cooperative Oncology Group.

^e^N/A: not assessed.

### Biopsy Procedure

Bone marrow biopsy was performed for all patients, and the median blood pressure was 141/79 mm Hg before and after biopsy for all patients, without a significant difference between the groups. Hypertension (≥140/90 mm Hg) was documented in 59.4% (63/118) of patients before biopsy (34/60, 63% in the MEOPA group, 29/58, 56% in the VR group; *P*=.45) and 55.4% (52/118) of patients 15 minutes after the biopsy (26/60, 57% in the MEOPA group and 25/58, 54% in the VR group; *P*=.83). A median of 1 vial of lidocaine was used for local anesthesia (range 1-3 vials); 24 patients received 2 or more vials of lidocaine (14 in the MEOPA group and 10 in the VR group; *P*=.17), and none received oral salvage treatment (Table S1 in Multimedia Appendix 1).

### Tolerance of the VR Session and Feeling Immersed

Patients enrolled in the VR group received a short demonstration of VR (5 minutes) to assess their tolerance. During the VR demonstration, most (57/59, 97%) of them watched the entire session, while 2 patients stopped the session: The first patient felt intolerance during the demonstration, and the second patient finally refused VR after completing the demonstration session without an adverse event (Table S2 in Multimedia Appendix 2).

For patients who completed the VR program during the biopsy, no adverse events occurred, and only 1 patient experienced a high level of anxiety and switched to MEOPA. Regarding sense of presence, 84% (47/56) of patients felt immersed during the VR session, 73% (41/56) felt elsewhere, and 66% (37/56) felt like they were in a dream (Table S3 in Multimedia Appendix 3).

### Pain Evaluation

Pain after biopsy was evaluated using 3 questionnaires: intensity with the VAS 15 minutes after the procedure, residual pain, and memory of pain 1 month after the procedure. In the intention-to-treat analysis with the last available information carried forward, the average pain was 3.5 (SD 2.6) and 3.0 (SD 2.4) in the MEOPA and VR groups, respectively (*P*=.27), and the median pain was 3.0 in both groups (Table 2). After 1 month, 104 patients answered the questionnaires on residual pain and memory of pain (52 patients per arm). The median residual pain assessed using the VAS was 0.0 for each group (range 0.0-3.0 for the MEOPA group and 0.0-5.0 for the VR group; *P*=.51). One-quarter (26/104, 25%) of the patients retained a memory of pain experienced during the biopsy at 1 month (15/52, 29% of the MEOPA group and 11/52, 21% of the VR group; *P*=.37), with a median score of 3.0 on the VAS (3.0 for the MEOPA group and 5.0 for the VR group; *P*=.31). There were no significant differences by age, group, sex, hemopathy, and centers.

**Table 2:** Pain intensity.

Pain intensity			Total (n=118)	MEOPA^a^ group (n=60)	VR^b^ group (n=58)	*P* value^c^	
**15 minutes after the biopsy**							
	Pain intensity, mean (SD)		3.3 (2.5)	3.5 (2.6)	3.0 (2.4)	.27	
	Pain, median (range)		3.0 (0-10)	3.0 (0-10)	3.0 (0-10)	N/A^d^	
	Pain, 95% CI		–1.6 to 8.2	–1.596 to 8.596	–1.704 to 7.704	N/A	
**1 month after the biopsy**							
	Completed questionnaires, n (%)		104 (88.1)	52 (86.7)	52 (89.7)	N/A	
	Residual pain, median (range)		0 (0-5)	0 (0-3)	0 (0-5)	.51	
	**Memory of pain, n (%)**						.37
		Yes	26 (25.0)	15 (28.9)	11 (21.1)		
		No	78 (75.0)	37 (71.1)	41 (78.9)		
	Evaluation of memory (analog visual scale), median (range)		3 (1-10)	3 (1-8)	5 (1-10)	.31	

^a^MEOPA: mixture of nitrous oxide/oxygen.

^b^VR: virtual reality.

^c^*t* test, except for memory of pain, which was assessed using a chi-square test.

^d^N/A: not assessed.

### Anxiety

Anxiety was assessed using 2 questionnaires: fear of pain and the revised STAI. The fear of pain questionnaire was completed by 117 patients (117/118, 99.1%). Among them, 67.5% (79/117) were afraid of the biopsy, with a median score of 5.0 (VAS from 0 to 10; 40 patients in the MEOPA arm and 39 patients in the VR arm). The causes for fear were the local injection of lidocaine in 40% (32/79) of cases, biopsy in 91% (72/79), and biopsy results in 73% (58/79; Table S4 in Multimedia Appendix 4).

The STAI questionnaire was completed by 114 patients. The theoretical range of scores is from 20 to 80 points: the higher the score, the more anxious the patient. The median score was 38 (range 20-73) and 32 (range 20-65) before and after the biopsy, respectively, for all patients, without any significant differences between the 2 groups (37 vs 39 before and 30 vs 33 after the biopsy for the MEOPA group vs VR group; *P*=.79 and *P*=.40, respectively). Of the patients, 26.3% (30/114) were anxious, with a score over 46, before the biopsy, and 9% (10/114) of patients were anxious after, without any significant difference between the MEOPA and VR groups (Table 3). After 1 month, 8.7% (9/104) of patients considered the biopsy to be psychologically traumatic (5/52 in the MEOPA group and 4/52 in the VR group; *P*>.99).

**Table 3:** Anxiety (Spielberger State-Trait Anxiety Inventory [STAI]) scores.

STAI scores				Total (n=114)		MEOPA^a^ group (n=57)		VR^b^ group (n=57)		*P* value^c^	
**Per arm, median (range)**											
	Before the biopsy		38 (20-73)		37 (20-73)		39 (20-69)		.79		
	After the biopsy		32 (20-65)		30 (20-65)		33 (20-57)		.40		
**Distribution of scores, n (%)**											
	**Before the biopsy**										.29
		Very low (<36)	41 (35.9)		23 (40.4)		18 (31.6)				
		Low (36-46)	43 (37.7)		19 (33.3)		24 (42.1)				
		Average (46-56)	17 (14.9)		6 (10.5)		11 (19.3)				
		High (56-65)	11 (9.6)		8 (14.0)		3 (5.2)				
		Very high (>65)	2 (1.8)		1 (1.8)		1 (1.8)				
	**After the biopsy**										.83
		Very low (<36)	73 (64.0)		39 (68.4)		34 (59.6)				
		Low (36-46)	28 (24.6)		12 (21.0)		16 (28.1)				
		Average score (46-56)	6 (5.3)		3 (5.3)		3 (5.3)				
		High (56-65)	4 (3.5)		2 (3.5)		2 (3.5)				
		Very high (>65)	0 (0)		0 (0)		0 (0)				
		Unknown	3 (2.6)		1 (1.8)		2 (3.5)				

^a^MEOPA: mixture of nitrous oxide/oxygen.

^b^VR: virtual reality.

^c^Chi-square test.

### Satisfaction

Patients, nurses, and physicians answered a satisfaction questionnaire 15 minutes after the biopsy (Table 4). The questionnaires were completed by 98.3% (116/118) of the patients. Physicians (n=24) completed 117 questionnaires (117/118, 99.1%), and nurses (total number of nurses not assessed) completed 116 questionnaires (116/118, 98.3%).

Satisfaction with the relaxation method was experienced by 98.3% (114/116) of patients, without a significant difference between the 2 groups (*P*=.45); the technique was recommended by 97% (56/58) of the MEOPA group and 90% (52/58) of the VR group (*P*=.13).

According to the nurses, the method was technically binding for 34.4% (40/116) of the cases (40/59, 24% of the MEOPA group and 26/57, 46% of the VR group; *P*=.03); pain was reported by 77.1% (43/116) of cases (20/59, 34% of the MEOPA group and 23/57, 40% of the VR group; *P*=.89), and stress was reported by physicians for 15.5% (18/116) of cases (9/59, 15% of the MEOPA group and 9/57, 16% of the VR group; *P*=.68). They observed crying in 6.9% (8/116) of patients (5/59, 9% of the MEOPA group and 3/57, 5% of the VR group; *P*=.71) and global satisfaction with the method in 95.7% (111/116) of cases (56/59, 95% of the MEOPA group and 55/57, 97% of the VR group; *P*=.80).

According to the physicians, MEOPA was easy to use in 78% (46/59) of cases, and VR was easy to use in 79% (46/58; *P*=.26) of cases. They recognized pain in 49.6% (58/117) of patients: 59% (35/59) of the MEOPA group and 40% (23/57) of patients in the VR group (*P*=.18). They felt relaxed during the biopsy of 88% (52/59) of cases in the MEOPA group and 93% (54/58) of cases in the VR group (*P*=.21). They were satisfied with the relaxation technique with 95.7% (112/117) of cases (54/59, 92% of the MEOPA group and 58/58, 100% of the VR group; *P*=.01) and recommended reuse of the method with 94.0% (110/117) of cases (53/59, 90% of the MEOPA group and 57/58, 98% of the VR group; *P*=.02).

**Table 4:** Satisfaction assessment.

Questions				Total (n=118) , n (%)		MEOPA^a^ (n=60), n (%)		VR^b^ (n=58) , n (%)		*P* value^c^	
**Physicians**											
	**Questionnaire completed?**										N/A^d^
		Yes	117 (99.1)		59 (98.3)		58 (100)				
		No	1 (0.9)		1 (1.7)		0 (0)				
	**Are you satisfied with the technique?**										.01
		Yes	112 (95.7)		54 (91.5)		58 (100)				
		No	5 (4.3)		5 (8.5)		0 (0)				
		If yes, a lot	76 (64.9)		31 (52.5)		45 (77.6)				
	**Did your patient experience pain?**										.18
		Yes	58 (49.6)		35 (59.3)		23 (39.7)				
		No	59 (50.4)		24 (40.7)		35 (60.3)				
		If yes, a lot	7 (6.0)		5 (8.4)		2 (3.5)				
	**Would you like to re-use the technique?**										.02
		Yes	110 (94.0)		53 (89.8)		57 (98.3)				
		No	7 (6.0)		6 (10.2)		1 (1.7)				
		If yes, a lot	78 (66.6)		32 (54.2)		46 (79.1)				
	**Was the technique restrictive?**										.26
		Yes	25 (21.4)		13 (22.1)		12 (20.7)				
		No	92 (78.6)		46 (77.9)		46 (79.3)				
		If yes, a lot	1 (0.9)		1 (1.7)		0 (0)				
	**Did you feel relaxed during the biopsy?**										.21
		Yes	106 (90.6)		52 (88.1)		54 (93.1)				
		No	11 (9.4)		7 (11.9)		4 (6.9)				
		If yes, a lot	60 (51.2)		25 (42.3)		35 (60.3)				
**Nurses**											
	**Questionnaire completed?**										N/A
		Yes	116 (98.3)		59 (98.3)		57 (98.3)				
		No	2 (1.7)		1 (1.7)		1 (1.7)				
	**Was the technique restrictive?**										.03
		Yes	40 (34.4)		14 (23.7)		26 (45.6)				
		No	76 (66.6)		45 (76.3)		31 (54.4)				
		If yes, a lot	2 (1.7)		1(1.7)		1 (1.8)				
	**Did the patient cry?**										.71
		Yes	8 (6.7)		5 (8.5)		3 (5.3)				
		No	108 (93.3)		54 (91.5)		54 (94.7)				
	**Did your patient experience pain?**										.89
		Yes	43 (77.1)		20 (33.9)		23 (40.4)				
		No	73 (32.9)		39 (76.1)		34 (59.6)				
		If yes, a lot	9 (7.8)		5 (8.4)		4 (7.0)				
	**Did you observe stress in the doctor?**										.68
		Yes	18 (15.5)		9 (15.2)		9 (15.8)				
		No	98 (84.5)		50 (84.8)		48 (84.2)				
	**Are you satisfied by the technique?**										.80
		Yes	111 (95.7)		56 (94.9)		55 (96.6)				
		No	5 (4.3)		3 (5.1)		2 (3.5)				
		If yes, a lot	55 (47.4)		28 (47.4)		27 (47.4)				
**Patients**											
	**Questionnaire completed?**										N/A
		Yes	116 (98.3)		58 (96.7)		58 (100)				
		No	2 (1.7)		2 (3.3)		0 (0)				
	**Are you satisfied with the technique?**										.45
		Yes	114 (98.3)		57 (98.3)		57 (98.3)				
		No	2 (1.7)		1 (1.7)		1 (1.7)				
		If yes, a lot	52 (44.8)		30 (51.7)		22 (37.9)				
	**Would you re-use the technique?**										.13
		Yes	108 (93.1)		56 (96.5)		52 (89.7)				
		No	8 (6.9)		2 (3.5)		6 (10.3)				
		If yes, a lot	49 (42.2)		29 (50.0)		20 (34.5)				

^a^MEOPA: mixture of nitrous oxide/oxygen.

^b^VR: virtual reality.

^c^Chi-square test.

^d^N/A: not assessed.

## Discussion

REVEH is the first randomized study to compare VR with standard MEOPA in preventing pain during bone marrow biopsy. The intensity of pain did not significantly differ between VR and MEOPA: 3.0 versus 3.5 (*P*=.27). Anxiety scores and blood pressure were not statistically different between the 2 arms, both before and after the biopsy. Patients, nurses, and physicians were very satisfied with the relaxation method, without significant differences between VR and MEOPA.

The main technical difficulty was related to the handling of the phone (connection and start of the VR program); a new generation of head-mounted displays has recently emerged with full smartphone integration to facilitate set-up and reduce start-up time (not investigated in this study). Only bone marrow biopsy was evaluated; the impact of VR in the same context should be evaluated for other procedures (eg, lumbar puncture, ascites puncture, pleural puncture, catheter placement). We do not know if the patients had already been exposed to VR during their leisure time (movies, video games; data not available). Patient choice of the scenario was not recorded in this study, so programs were not compared with each other. Patients also could not compare them from one procedure to another (only 1 biopsy per patient). Pain and anxiety are very subjective symptoms that vary from patient to patient and are influenced by the self-experience. Physiological markers (clinical, biological parameters, neuroimaging markers) to objectively assess pain and anxiety have been published: blood pressure and heart rate variations, pupil reflexes, stress hormonal changes, electro-encephalography, magnetic resonance imaging, and positron emission tomography to assess brain activity [30]. These parameters could be evaluated in a randomized study comparing all distraction methods (VR, hypnosis, music, television, video games).

Even if the primary outcome of the study has not been achieved, the VR-based relaxation method was well tolerated, and the satisfaction of patients and physicians was very high in the VR group. In the future, the patient will put the helmet on himself or herself and choose his or her own program; in contrast to the use of analgesics or sedatives, no medical monitoring is indicated during and after the procedure, and driving is allowed. The length of time that the patient must be present will be shorter, with a probable socioeconomic impact, which will have to be evaluated.

Several meta-analyses were published between 2019 and 2020 that evaluated the impact of VR on fatigue, anxiety, depression, and care-induced pain [31-34]. Based on the PRISMA (Preferred Reporting Items for Systematic Reviews and Meta-Analyses) guidelines, 67 studies were selected for analysis (6/293 screened for Zeng et al [31], 20/4415 screened for Eijlers et al [32], 18/1589 screened for Smith et al [33], and 23/838 screened for Ioannou et al [34]). The studies were heterogeneous in terms of primary endpoint (pain, anxiety, fatigue, depression, cognitive function), study population, number of patients per study (maximum 143 patients), age, underlying pathology, type of study (randomized or not, crossover allowed), and standard arm. Regarding pain and anxiety, VR significantly reduced scores in 74% and 63% of the studies, respectively [31-35]. Effectiveness was particularly significant in very young children [32], but the effects varied according to the indication, content of the environment, type of program (active vs passive), number of sessions and complete duration of the VR experience, and patient populations (cancer patients vs noncancer patients) [33].

### Conclusion

Pain intensity did not significantly differ between VR and MEOPA arms during bone marrow biopsy in this study. The VR-based distraction method was safe and appreciated by patients and caregivers. Digital therapeutics could be an alternative treatment in case of contraindication or intolerance to MEOPA and could be integrated into the oncology support care panel.

Further studies should focus on demonstrating the efficacy of a VR program (content, type, interactions, number of sessions, program duration) in pain prevention and other symptoms (fatigue, depression, anxiety, cognitive functions) for each indication, compared with conventional drugs with a noninferiority outcome, if possible, and based on feasible objective parameters. The impact of VR will also have to be studied for patients hospitalized for a long period of time and at home for outpatients. For chronic pain, VR should be evaluated in terms of scheduling, efficacy, and tolerability to reduce opioid dependence in cancer patients [36-39]. Pharmacoeconomic studies must also be supported.

## Appendix

Multimedia Appendix 1 Bone marrow events.

Multimedia Appendix 2 VR tolerance (n=59).

Multimedia Appendix 3 Feeling of immersion (n=56).

Multimedia Appendix 4 Fear of pain.

Multimedia Appendix 5 CONSORT-eHEALTH checklist V 1.6.1.

## Abbreviations

ECOG: Eastern Cooperative Oncology Group
MEOPA: mixture of nitrous oxide/oxygen
PRIMSA: Preferred Reporting Items for Systematic Reviews and Meta-Analyses
STAI: Spielberger State-Trait Anxiety Inventory
VAS: visual analog scale
VR: virtual reality

## Data Availability

All data from the study are freely available upon request to the corresponding author.
