# Supplementary material for: A New Option for Pain Prevention Using a Therapeutic Virtual Reality Solution for Bone Marrow Biopsy (REVEH Trial): Open-Label, Randomized, Multicenter, Phase 3 Study
Source: J Med Internet Res. 2023 Feb 15;25:e38619. doi: 10.2196/38619 (PMC9978987; doi:10.2196/38619)
Supplement: Multimedia Appendix 1 [file jmir_v25i1e38619_app1.docx]

**Table S1. Bone marrow events.**

| Bone marrow biopsy | TOTAL  n= 118 | MEOPA  n= 60 | VR  n= 58 | *P* value  (Chi^2^test except for blood pressure: Wilcoxon test)) |
| --- | --- | --- | --- | --- |
| Median systolic arterial blood pressure (range) |  |  |  |  |
| Before the biopsy | 141.0 (92-203) | 141.5 (101-186) | 140.0 (92-203) | .470 |
| 15 minutes after | 140.5 (103-191) | 141.5 (103-190) | 140.0 (107-191) | .827 |
| Median diastolic arterial blood pressure (range) |  |  |  |  |
| Before the biopsy | 79.5 (52-109) | 78 (60-100) | 80 (52-109) | .693 |
| 15 minutes after  Hypertension (≥ 140/90)  Before the biopsy  After the biopsy | 79 (58-113)  63 (59.4)  52 (55.4) | 78.5 (58-113)  34 (63.0)  26 (56.5) | 79.5 (64-98)  29 (55.8)  25 (54.3) | .711  .450  .834 |
| Procedure discontinuation n (%) |  |  |  | >.990 |
| Yes | 2 (1,7) | 1 (0,8) | 1 (0.8) |  |
| No | 115 (98.2) | 58 (99.9) | 57 (99.9) |  |
| Number of vials n (%) |  |  |  | .166 |
| 1 | 93 (79.5) | 45 (76.3) | 48 (82,8) |  |
| 2 | 22 (18.8) | 14 (23.7) | 8 (13.8) |  |
| 3 | 2 (1.7) | 0 | 2 (3.4) |  |
| Time MEOPA exposure (min, range) | - | 10 (0-20) | - |  |
|  |  |  |  |  |

(VR: virtual reality, MEOPA: mixture of nitrous oxyde/oxygen)
