# Supplementary material for: A New Option for Pain Prevention Using a Therapeutic Virtual Reality Solution for Bone Marrow Biopsy (REVEH Trial): Open-Label, Randomized, Multicenter, Phase 3 Study
Source: J Med Internet Res. 2023 Feb 15;25:e38619. doi: 10.2196/38619 (PMC9978987; doi:10.2196/38619)
Supplement: Multimedia Appendix 2 [file jmir_v25i1e38619_app2.docx]

**Table S2. VR tolerance (n=59).**

| Demonstration session performed before biopsy, n (%) |  |
| --- | --- |
| No | 2 (3.4) (1: refusal to continue without adverse event, 1: intolerance and switch for MEOPA) |
| Yes | 57 (96.6) |
| Events during biopsy with VR, n (%) | 57 evaluable patients |
| Nausea |  |
| No | 57 (100) |
| Dizziness |  |
| No | 57 (100) |
| Headache |  |
| No | 57 (100) |
| Session stopped |  |
| No | 54 (94.7) |
| Yes | 3 (5.3) (1: temporary interruption for an uncomfortable position, 1: session restart, |
|  | 1: unbearable anxiety, switch to MEOPA) |

(MEOPA, mixture of nitrous oxyde/oxygen, VR, virtual reality)
