# Supplementary material for: A New Option for Pain Prevention Using a Therapeutic Virtual Reality Solution for Bone Marrow Biopsy (REVEH Trial): Open-Label, Randomized, Multicenter, Phase 3 Study
Source: J Med Internet Res. 2023 Feb 15;25:e38619. doi: 10.2196/38619 (PMC9978987; doi:10.2196/38619)
Supplement: Multimedia Appendix 3 [file jmir_v25i1e38619_app3.docx]

**Table S3. Feeling of immersion (n=56).**

| **Did you feel immersed in VR environment?** |  |
| --- | --- |
| not at all | 9 (16.1) |
| a little | 13 (23.2) |
| enough | 23 (41.1) |
| very | 11 (19.6) |
|  |  |
| **Did you feel elsewhere?** |  |
| not at all | 15 (26.8) |
| a little | 17 (30.3) |
| enough | 16 (28.6) |
| very | 8 (14.3) |
|  |  |
| **Did you feel like in a dream?** |  |
| **or did you have the feeling of travelling?** |  |
| not at all | 19 (33.9) |
| a little | 17 (30.4) |
| enough | 11 (19.6) |
| very | 9 (16.1) |
|  |  |

(VR: virtual reality)
