# Supplementary material for: A New Option for Pain Prevention Using a Therapeutic Virtual Reality Solution for Bone Marrow Biopsy (REVEH Trial): Open-Label, Randomized, Multicenter, Phase 3 Study
Source: J Med Internet Res. 2023 Feb 15;25:e38619. doi: 10.2196/38619 (PMC9978987; doi:10.2196/38619)
Supplement: Multimedia Appendix 4 [file jmir_v25i1e38619_app4.docx]

**Table S4. Fear of pain.**

| Fear of pain | TOTAL  n= 117 | MEOPA group  n= 59 | VR group  n= 58 | p value |
| --- | --- | --- | --- | --- |
| Fully completed questionnaire: n (%) | 117 (100) | 59 (100) | 58 (100) |  |
| Presence of fear before biopsy |  |  |  | .948 |
| No | 38 (32.5) | 19 (32.2) | 19 (32.8) |  |
| Yes | 79 (67.5) | 40 (67.8) | 39 (67.2) |  |
| Fear intensity (visual analogic scale) |  |  |  | .171 |
| Median (range) | 5 (0-10) | 5 (1-10) | 5 (0-10) |  |
| Fear of local injection with lidocaine: n (%) |  |  |  | .917 |
| No | 47 (59.5) | 24 (60) | 23 (59) |  |
| Yes a little | 20 (25.3) | 11 (27.5) | 9 (23.1) |  |
| Yes moderately | 9 (11.4) | 4 (10) | 5 (12.8) |  |
| Yes intensely | 3 (3.8) | 1 (2.5) | 2 (5.1) |  |
| Fear of bone marrow biopsy: n (%) |  |  |  | .361 |
| No | 7 (8.9) | 2 (5) | 5 (12.8) |  |
| Yes a little | 34 (43) | 16 (40) | 18 (46.2) |  |
| Yes moderately | 25 (31.7) | 13 (32.5) | 12 (30.8) |  |
| Yes intensely | 13 (16.5) | 9 (22.5) | 4 (10.2) |  |
| Fear of biopsy results: n (%) |  |  |  | .497 |
| No | 21 (26.6) | 9 (22.5) | 12 (30.8) |  |
| Yes a little | 21 (26.6) | 12 (30) | 9 (23.1) |  |
| Yes moderately | 23 (29.1) | 10 (25) | 13 (33.3) |  |
| Yes intensely | 14 (17.7) | 9 (22.5) | 5 (12.8) |  |
|  |  |  |  |  |

(VR: virtual reality, MEOPA: mixture of nitrous oxide/oxygen)
